# Age-dependent alterations in the inflammatory response to pulmonary challenge

**DOI:** 10.1007/s12026-015-8684-7

**Published:** 2015-08-29

**Authors:** Helena M. Linge, Kanta Ochani, Ke Lin, Ji Young Lee, Edmund J. Miller

**Affiliations:** Center for Heart and Lung Research, The Feinstein Institute for Medical Research, 350 Community Drive, Manhasset, NY 11030 USA; Departments of Molecular Medicine, Medicine and Surgery, Hofstra North Shore-LIJ School of Medicine, Hempstead, NY 11549 USA

**Keywords:** Pneumonia, Aging, Thyroxine, Acute lung injury

## Abstract

The aging lung is increasingly susceptible to infectious disease. Changes in pulmonary physiology and function are common in older populations, and in those older than 60 years, pneumonia is the major cause of infectious death. Understanding age-related changes in the innate and adaptive immune systems, and how they affect both pulmonary and systemic responses to pulmonary challenge are critical to the development of novel therapeutic strategies for the treatment of the elderly patient. In this observational study, we examined age-associated differences in inflammatory responses to pulmonary challenge with cell wall components from Gram-positive bacteria. Thus, male Sprague–Dawley rats, aged 6 months or greater than 18 months (approximating humans of 20 and 55–65 years), were challenged, intratracheally, with lipoteichoic acid and peptidoglycan. Cellular and cytokine evaluations were performed on both bronchoalveolar lavage fluid (BAL) and plasma, 24 h post-challenge. The plasma concentration of free thyroxine, a marker of severity in non-thyroidal illness, was also evaluated. The older animals had an increased chemotactic gradient in favor of the airspaces, which was associated with a greater accumulation of neutrophils and protein. Furthermore, macrophage migration inhibitory factor (MIF), an inflammatory mediator and putative biomarker in acute lung injury, was increased in both the plasma and BAL of the older, but not young animals. Conversely, plasma free thyroxine, a natural inhibitor of MIF, was decreased in the older animals. These findings identify age-associated inflammatory/metabolic changes following pulmonary challenge that it may be possible to manipulate to improve outcome in the older, critically ill patient.

## Introduction

In normal individuals, the maximum functional ability of the lungs occurs between the ages of 20–25 years, after which there is a progressive decline in performance [1]. The changes are mainly associated with decreases in static elastic recoil, compliance of the chest wall, and in the strength of respiratory muscles. In later life, the susceptibility to lung infection also increases, and pneumonia occurs in around 25–44 cases per 1000 non-institutionalized elderly individuals [2], and in those older than 65 years old, pneumonia is the major cause of infectious death [3]. Studies of critically ill patients have identified advanced age and pulmonary staphylococcal infection as leading causes of sepsis [4]. Acute lung injury (ALI) or the adult respiratory distress syndrome (ARDS), as it is now more formally known in humans [5], is a major complication of nosocomial pneumonia [6] and is associated with acute inflammation leading to disruption of the lung epithelial and endothelial barriers. The inflammation is associated with greatly increased accumulation of neutrophils and other inflammatory cells induced by increased chemotaxins within the alveolae [7–9]. The injury is also associated with increased accumulation of the proinflammatory mediator macrophage migration inhibitory factor (MIF) within the airspaces [10]. MIF has been shown to be released by the lungs, into the systemic circulation, where it induces cardiocirculatory depression [11]. Because of its importance in acute inflammation, several groups have developed inhibitory antibodies [12–14] and synthetic small molecule inhibitors of MIF [15–18], and shown that they can prevent morbidity and mortality associated with endotoxemia and sepsis [16, 18–20]. Interestingly, MIF has the ability to override the anti-inflammatory activity of glucocorticoids [21], which, although their use remains controversial, have been used as therapeutic agents in the treatment for ARDS [22–24]. Furthermore, recent studies have shown that the thyroid hormone thyroxine, which is often decreased in critically ill patients, even in the absence of thyroidal illness [25], is a natural inhibitor of the proinflammatory active site of MIF [26].

In this observational study, components of the cell wall of Gram-positive bacteria, such as *S*. *aureus*, were used to incite an acute lung inflammation. We then evaluated age-dependent changes in the inflammatory response, in both the blood and alveolar compartments, at 24 h following the insult.

## Methods

### Animals Studies

All animal experiments received prior approval by the Institutional Animal Care and Use Committee of The Feinstein Institute. Male Sprague–Dawley rats (4 or 12 months of age) were purchased from Charles River (Kingston, NY). The latter were housed under standard conditions and provided with standard rodent chow and water, until experimentation at 18–20 months of age.

### Experimental Protocol

Three groups were investigated. Young (<6 months saline; <6 months LTA + PGN) and old (>18 months LTA + PGN). Each rat (*n* = 6 per group) was weighed and then anesthetized to a surgical plane using isoflurane. The trachea was then surgically exposed and cell wall components from Gram-positive bacteria lipoteichoic acid (0.15 mg) and peptidoglycan (0.5 mg), or saline alone in 100 ml sterile 0.9 % saline, was instilled intratracheally using a 29-gauge needle. The wound was then closed using 5.0 silk sutures, and the animals were allowed to recover. Once ambulant, they were returned to normal housing and allowed food and water ad libitum. After 24 h, the animals were re-anesthetized with isoflurane and exsanguinated via cardiac puncture, and the blood was collected for analysis. *Postmortem*, the lungs were lavaged with two aliquots (7 ml each) of sterile saline (0.9 %w/v). The bronchoalveolar lavage fluid (BAL) from each lavage was collected in separate tubes. The tubes were centrifuged, and supernatant was stored in −80 °C. Only the supernatant from the first tube was used for measuring chemokines, cytokines, and protein content, whereas BALF from both tubes was used for cell counting [27].

### Analysis of Bronchoalveolar Lavage Fluid

BAL cells were mixed with 0.5 ml of 0.2 % saline for 10 s to lyse any residual erythrocytes. The cells were then resuspended in 10 ml Hank’s buffered salt solution (HBSS). Total cell number was determined with a hemocytometer. Slides were prepared using a Shandon cytocentrifuge (Shandon Scientific, London, UK), and cells were stained with Wright-Giemsa stains (HEMA 3 Stain Set, Fisher Scientific, Pittsburgh, PA) for differential cell counting. Counts were made on at least 200 cells per slide.

Protein concentrations in BAL were assessed by BCA method (Pierce, Rockford, IL).

Commercially available enzyme-linked immunosorbent assay (ELISA) kits were used to assay MIF and KC (MyBioSource, San Diego, CA) and free thyroxine (Genway Biotech, Inc., San Diego, CA) following the manufacturers’ instructions.

### Statistical Analysis

Data are presented as mean ± standard deviation. One-way ANOVA was used to evaluate the statistical significance of the results. Difference with *p* value < 0.05 was considered significant.

## Results

### Body Weight Increased with Age

**Fig. 1:**
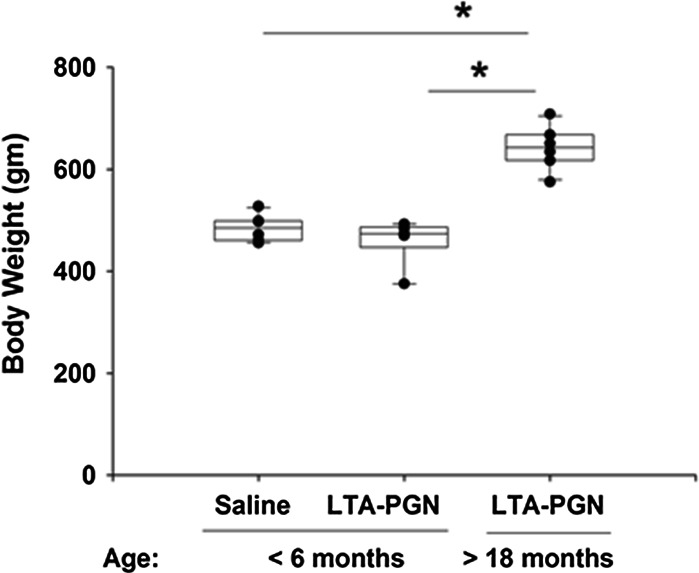
Body weights prior to pulmonary challenge. The weights of the older animals were significantly greater than those of the younger group (*p < 0.05)

Sprague–Dawley rats can live up to 3.5 years, and males reach an adult body weight of between 450 and 520 g around 12 weeks old. In the current study, the body weights of the rats were 473 ± 38 g in the <6-month group and 641 ± 45 g in the >18-month group (Fig. 1). However, in the current study, animals were challenged with the same amount of LTA and PGN, and it was not titered with respect to body weight.

### Polymorphonuclear Leukocyte (Neutrophil) Influx into the Airspaces

**Fig. 2:**
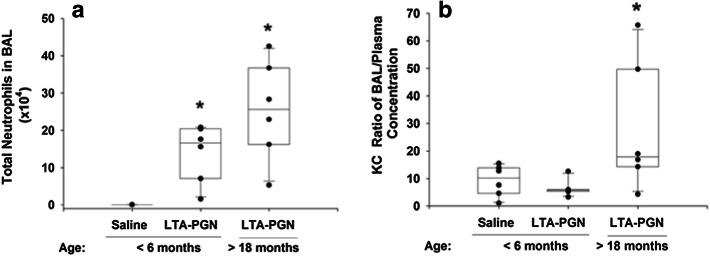
Neutrophil accumulation in the airspaces of the lungs **a** was significantly greater in the rats challenged with LTA and PGN than in controls. **b** The ratio of the KC concentration in the BAL and Plasma KC was significantly higher in the >18-month group than in either of the other two groups (*p < 0.05)

Neutrophils accumulate at a site of inflammation by responding to a chemotactic gradient. In rats, KC (CXCL1) and its cellular receptor CXCR2 have been shown to be a major chemokine for the pulmonary recruitment of neutrophils [28]. Therefore, we measure the concentration of this chemokine in both plasma and the airspaces, and assessed its association with the accumulation of neutrophils within the alveolae. Following pulmonary challenge, there was a significant increase (*p* = 0.05) in the percentage of neutrophils in the blood of the <6-month group (69 ± 13 %) compared to controls (39 ± 10 %). However, in the older group >18 months, there was no significant increase in the percentage of blood neutrophils (42 ± 14 %). Concurrently, in the BAL, the total number of neutrophils significantly increased from control values in both groups of rats treated with LTA–PGN (Fig. 2a). The neutrophilic influx was associated with a significant accumulation of KC within the airspaces of the older animals (43.8 ± 18.5 ng/ml) compared to controls (16.5 ± 4.0) and <6-month group (21.6 ± 6.6). However, this was not reflected in the plasma accumulation of KC controls (4.2 ± 5.9 ng/ml); <6 months (3.7 ± 1.4); >18 months (2.3 ± 1.5). Since neutrophil chemotaxis depends on the movement of cells along a chemotactic gradient, we assessed the ratio of the concentration of KC in the BAL and plasma (Fig. 2b). While there was no significant difference between the younger groups, in this respect, the >18 months was significantly greater than the other two groups. It should be noted that BAL is approximately 100-fold more dilute than the epithelial lining fluid that it samples [7]. Thus, there is a considerable chemotactic gradient between the blood and alveolar spaces in the LTA–PGN challenged animals. The influx of neutrophils was associated with significantly increased accumulation of protein (*p* < 0.05) in the <6-month group (427 ± 208 μg/ml) and >18-month-old group (584 ± 297 μg/ml) within the alveolar space of challenged animals compared to controls (108 ± 40 μg/ml), suggesting that some injury to the airspaces had occurred in each of the challenged groups.

### Macrophage Migration Inhibitory Factor Accumulation

**Fig. 3:**
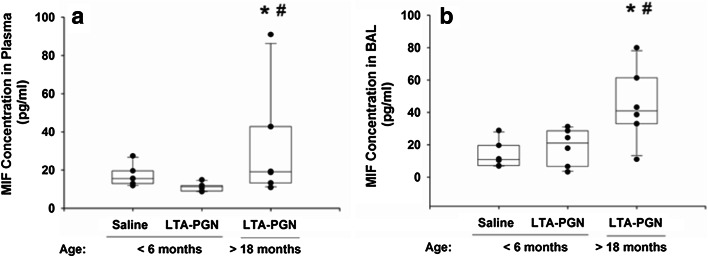
MIF concentration in both the plasma (**a**) and the airspaces (**b**) was significantly greater in the older animals than in controls (*p < 0.05) or the younger animals following an identical pulmonary challenge (^#^ *p* < 0.05)

While MIF is found within the airspaces of normal lungs, there is a significant increase in accumulation in the alveolar airspaces of patients with ARDS [10]. In addition, animal studies suggest that there is enhanced MIF expression in the alveolar endothelium and infiltrating macrophages in ARDS [14, 29], and that the lungs may act as an inflammatory organ releasing MIF into the systemic circulation [30], where it can induce cardiocirculatory depression [11]. Therefore, we examined the concentration of MIF in blood and BAL following pulmonary challenge. At 24 h post-challenge, there was only an increase in MIF of the plasma of the >18-month-old group (Fig. 3a). However, a significant increase in plasma MIF was only see in two (33 %) of the animals. A more profound increase in MIF concentration was noted within the airspaces of the older group (Fig. 3b).

### Free Thyroxine Evaluation

**Fig. 4:**
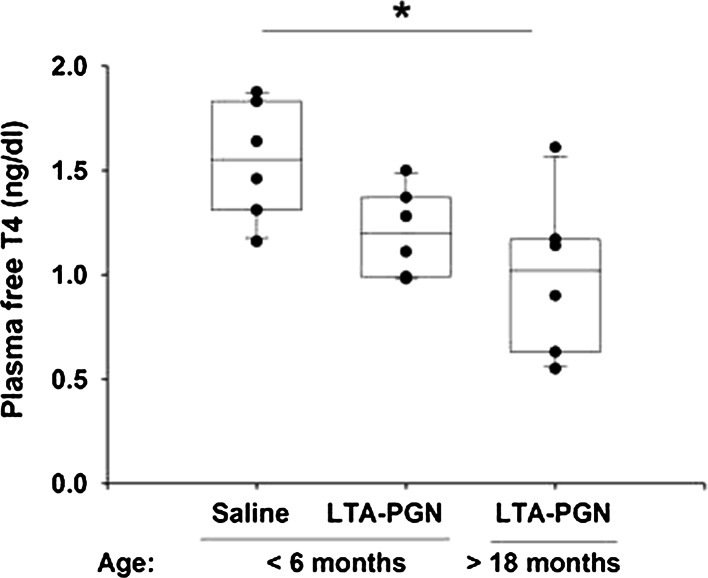
Plasma free thyroxine (fT4) was significantly decreased in the older animals 24 h post-pulmonary challenge (*p < 0.05)

A characteristic finding in patients with critical illness is an alteration in circulating thyroid hormone levels including thyroxine (T4). The changes in thyroid hormone concentrations are directly correlated to the severity of the disease and are associated with poor survival [31]. Accordingly, we measured plasma free T4 in the rats following pulmonary challenge (Fig. 4). The median free T4 was significantly lower in the older age group (1.02 ng/dl) than the controls (1.55 ng/dl). However, there was no difference between the young group (1.20 ng/dl) and controls.

## Discussion

The ability of the body to mount a concerted response is an evolutionary advantage. In the case of bacterial challenges, our body is tested by a diverse, complex and dynamic challenge, and must adapt its constitutive functions to repel the threat. In many cases, the response to the challenge is appropriate and homeostasis maintained. However, there are other situations in which the body’s overall inflammatory response is seemingly inappropriate, lacking effectiveness, and leads to detrimental effects and even death.

Infection by the Gram-positive bacteria *Staphylococcus aureus* or *Streptococcus pneumonia* is a common cause of pneumonia, especially in elderly patients [32–34]. The elderly are at increased risk of pneumonia due to age-related deterioration of lung function [35]. Studies of critically ill patients have identified advanced age and pulmonary *S. aureus* infection as leading causes of sepsis [4]. During the progression of infection and pneumonia, ALI can develop. ALI is a major clinical condition which affects nearly 200,000 individuals annually in the USA [36] and a major complication of nosocomial pneumonia [37]. The clinical manifestations include severe hypoxemia with acute onset and bilateral infiltrates on chest radiography [38]. Although effective ventilator and fluid management strategies have been developed, mortality remains high at 25–35 % [39–41]. Age is a critical determinant of the incidence and mortality of ALI [incidence: 16 per 100,000 person-years in young (15–19 years of age) and 306 per 100,000 person-years (75–84 years of age) mortality: 24 % (15–19 years) to 60 % (>85 years)] [36]. Critically ill patients with ALI or its most severe form ARDS, have an increased capillary permeability, which results in significant fluid imbalance, oncotic pressure changes, pulmonary edema, and alveolar collapse [42]. While comorbidities such as chronic obstructive pulmonary disease (COPD) and cardiovascular disease are major risk factors for pneumonia in the elderly, it is also clear that there are changes in the inflammatory response even in the healthy individual [43]. While studies show that pro-inflammatory cytokines in blood and tissues are higher than in their young counterparts [44, 45], this may not be the complete, or indeed the fundamental, reason for the increased mortality. Thus, it remains unclear whether there are alterations in magnitude of the response or whether the normally highly orchestrated sequence of events is dysregulated. The sequence of events that occur in the inflammatory response is critical to the effectiveness of that response.

Animal studies involving older animals are expensive and often more difficult to perform due to the “fragility” of the animal with greater morbidity and mortality associated with this group. Also, as in the current study, the variability of the responses in the older animals is greater than their younger counterparts. Therefore, many of the studies involving acute lung inflammation, ALI, and sepsis are performed in young animals, and there is much less information about the pathophysiology of acute inflammation, which is specific to older patients [46]. However, studies have shown important age-associated increase in mortality, hypothermia, and induction of IL-6 following Gram-negative or [47] Gram-positive [48] challenge. While the current study only examines one dose of bacterial toxin and one time point of sampling, it highlights some pertinent differences between the groups and changes that occur following pulmonary challenge in a rat model of acute inflammation, and the differing response to an identical challenge in the older animal group. First, the body weights of the older age group, prior to challenge, were significantly greater than controls. Starr et al. [49] have shown that white adipose tissue is a major source of interleukin-6 (IL-6), a proinflammatory cytokine mediating the severity of sepsis [50]. Furthermore, obesity is associated with an increased number of activated circulating neutrophils [51]. Previous studies have shown that activated neutrophils have a longer life span than their quiescent counterparts [52]. Interestingly, 24 h post-challenge, there was no change in the percentage of neutrophils in the blood of the older group, but there were more neutrophils in the airspaces. The increased accumulation was associated with a more profound chemotactic gradient, in favor of the airspaces, in the older animals (Fig. 2). This suggests that in the younger animals, the neutrophil recruitment had terminated earlier than in the older group. Perhaps more importantly, the inflammatory effects of the chemokine may be more profound in the older group. This is because KC and its human counterpart Interleukin-8 not only induce neutrophil chemotaxis, but at higher concentrations initiate neutrophil degranulation and the generation of hydrogen peroxide and superoxide anion [28, 53]. Of note, elevated levels of interleukin-8 are present in the airspaces of patients with the adult respiratory distress syndrome and are associated with increased mortality [7].

We also found increased accumulation of MIF in both the plasma and airspaces of the older animals 24 h post-challenge. MIF is a relatively small protein mediator produced by numerous cell types, including alveolar macrophages [30], neutrophils [31], pulmonary epithelial cells [32], and pulmonary endothelial cells [32, 33]. During severe infection, MIF accumulates both in the parenchymal tissue and in the alveolar spaces. The intra-alveolar MIF interacts with CD74 expressed on the surface of alveolar macrophage cells, inducing p44/p42 MAPK activation and chemokine release [35]. Furthermore, the MIF can be released into the systemic circulation where it induces cardiocirculatory depression [34, 36]. Notably, the increase in MIF was concurrent with a significant decrease in free T4 (Fig. 4). T4 has been shown to be a potent natural inhibitor of MIF, and that plasma MIF and fT4 concentrations are inversely correlated in patients with severe sepsis [26]. The study also showed that in thyroid-deficient animals, the outcome from severe infection is worse than euthyroid animals, and that inhibition of MIF with the hormonally inactive D-isomer of T4 protects against mortality. Furthermore, Ma et al. [54] have shown that acute lung injury is associated with an upregulation of type 2 deiodinase (DIO2) which metabolizes T4 into triiodothyronine (T3), a molecule involved in surfactant homeostasis and lung compliance [55, 56]. Thus, there may be many reasons for the low fT4 during acute lung injury. However, reduced availability of T4 may have profound downstream effects on lung function. This may be of particular importance to the older population, in whom an existing mild or subclinical hypothyroidism is more common [57], may also worsen many risk factors [58], and be directly related to the severity of acute illness [31].

## Summary

Changes in pulmonary physiology, pathology, and function are common in older populations, and lead to altered responses and increased morbidity and mortality associated with lung infections. However, in many respects, the reasons for the altered susceptibility remain unclear. There may be interactions between inflammatory and metabolic pathways, such as with MIF and T4, or T3 and surfactant homeostasis, that are not immediately obvious. While the benefit of direct supplementation of thyroid hormones in patients with critical illnesses such as ARDS or sepsis remains unclear [59], perhaps addressing the possible causes of the deficiency may prove more effective, as they have in animal models [11, 26, 54, 60, 61]. What is clear is that a better understanding of age-related changes in the innate and adaptive immune systems within the pulmonary compartment, and their impact on lung host defense, is of critical importance for developing effective therapeutic strategies for the treatment of the elderly patient.
